# A longitudinal analysis of nosocomial bloodstream infections among preterm neonates

**DOI:** 10.1007/s10096-022-04502-8

**Published:** 2022-09-30

**Authors:** Sophie J. Jansen, Alieke van der Hoeven, Thomas van den Akker, Marieke Veenhof, Erik G. J. von Asmuth, Karin Ellen Veldkamp, Monique Rijken, Martha van der Beek, Vincent Bekker, Enrico Lopriore

**Affiliations:** 1 Division of Neonatology, Department of Pediatrics, Willem Alexander Children’s Hospital, Leiden University Medical Center (LUMC), Leiden, The Netherlands; 2 Department of Medical Microbiology, Leiden University Medical Center (LUMC), Leiden, The Netherlands; 3 Department of Obstetrics & Gynecology, Leiden University Medical Center (LUMC), Leiden, The Netherlands; 4 Department of Pediatrics, Willem Alexander Children’s Hospital, Leiden University Medical Center (LUMC), Leiden, The Netherlands

**Keywords:** Neonates, Hospital-acquired infections, Central-lines, Epidemiology

## Abstract

Nosocomial bloodstream infections (NBSIs), commonly due to central-line associated bloodstream infections (CLABSI), contribute substantially to neonatal morbidity and mortality. We aimed to identify longitudinal changes in incidence of NBSI, microbiological-spectrum, and antibiotic exposure in a large cohort of preterm neonates admitted to the neonatal intensive care unit. We retrospectively assessed differences in annual rates of NBSI (per 1000 patient-days), CLABSI (per 1000 central-line days), and antibiotic consumption (per 1000 patient-days) among preterm neonates (< 32 weeks’ gestation) hospitalized between January 2012 and December 2020. Multi-state Markov models were created to model states of progression of NBSI and infection risk given a central-line on days 0, 3, 7, and 10 of admission. Of 1547 preterm infants, 292 (19%) neonates acquired 310 NBSI episodes, 99 (32%) of which were attributed to a central-line. Over the years, a significant reduction in central-line use was observed (*p* < 0.001), although median dwell-time increased (*p* = 0.002). CLABSI incidence varied from 8.83 to 25.3 per 1000 central-line days, with no significant difference between years (*p* = 0.27). Coagulase-negative staphylococci accounted for 66% of infections. A significant decrease was found in antibiotic consumption (*p* < 0.001). Probability of NBSI decreased from 16% on day 3 to 6% on day 10. NBSI remains a common problem in preterm neonates. Overall antibiotic consumption decreased over time despite the absence of a significant reduction in infection rates. Further research aimed at reducing NBSI, in particular CLABSI, is warranted, particularly with regard to limiting central-line dwell-time and fine-tuning insertion and maintenance practices.

**Supplementary Information:**

The online version contains supplementary material available at 10.1007/s10096-022-04502-8.

## Introduction

Hospital-acquired infections (HAIs) in neonates are a leading cause of neonatal morbidity and mortality [1]. Over the past two decades, the incidence of HAI has increased concurrently with improved survival of preterm infants, suggesting that hospitalization and use of invasive medical devices are important contributing factors [2, 3]. Nosocomial bloodstream infections (NBSIs), including central-line associated bloodstream infections (CLABSIs), are the most common type of HAI in neonates admitted to the neonatal intensive care unit (NICU). Prematurity, extended respiratory support, and previous antibiotic exposure are primary determinants of susceptibility for HAI and detrimental long-term (neurodevelopmental) outcomes [4, 5, 6]. Despite the extensive number of resources expended on eradicating HAIs, progress in tackling NBSI in neonates has been slow, pointing towards the need for increased efforts to eliminate this iatrogenic harm.

Given that HAIs are potentially preventable, monitoring the epidemiology of HAIs is essential to identify pathways of disease, risk factors, and areas for quality improvement, including an assessment of targeted interventions and changes in clinical practice intended to reduce HAIs. Moreover, evaluation of trends in antimicrobial use in the context of varying infection levels is necessary to inform future antibiotic stewardship efforts. As such, the present study was undertaken to determine longitudinal changes in the incidence of laboratory-confirmed NBSI, pathogen distribution, and overall antibiotic use in preterm neonates admitted to the NICU over a 9-year surveillance period, and to identify areas for quality improvement.

## Materials and methods

### Setting

The Leiden University Medical Center (LUMC) is the national referral center in the Netherlands for fetal therapy and pediatric cardiothoracic surgery, receiving high-risk pregnant women and patients from other (referral) centers. The hospital contains a 25-bed, level-III NICU comprised of 17 single-family rooms and 4-twin rooms, with average annual admission rate of 600–650 neonates. Average nurse-to-patient ratio is 1:1 to 1:2, depending on dependency levels. Parents are encouraged to stay with their newborn and permitted unrestricted access. Prior to construction of the single-room facility in May 2017, the NICU consisted of three open-bay wards, two of which served as intensive care units and one as high-care unit, with a total of 25 beds.

### Subjects and data collection procedures

This single-center, retrospective cohort study comprised all neonates born below 32 weeks of gestation admitted to the NICU between January 1st, 2012 and December 31st, 2020. Neonates were excluded if (i) duration of admission was shorter than 24 h due to early death, immediate transfer or ambulatory care, or (ii) admitted for (pre)terminal comfort care (defined as abstaining from intubation and cardiac resuscitation). In accordance with Dutch national policy to offer invasive intensive care from a gestational age of 24^0/7^ weeks onward [7], neonates born below this limit were also excluded. For infants with one or more readmissions, only data pertaining to the first admission were included. Approval and a waiver regarding written informed consent were obtained from the institutional review board of LUMC (no. G20.096).

Data on patients’ characteristics including birth date, admission date, gestational age, birth weight, sex, mode of birth (i.e., caesarian section or vaginal birth), 5-min Apgar score, presence of major congenital anomalies, exposure to invasive mechanical ventilation, duration of invasive mechanical ventilation, exposure to antibiotic therapy ≤ 24 h postpartum and ≥ 72 h after admission, length of antibiotic treatment, length of hospital stay, and in-hospital neonatal mortality were collected from electronic medical records spanning the period between admission and hospital discharge. Information on NBSI included microbiology (organisms isolated from clinical cultures), laboratory results (C-reactive protein, CRP), and source of infection. CLABSI data included presence of central-line including type (i.e., umbilical-venous, umbilical-arterial, or peripherally inserted central venous catheter), number of central-lines per neonate, duration of central-line use, and isolated pathogenic microorganism(s) in case of a confirmed CLABSI.

### Definitions

Diagnosis of NBSI was based on a positive blood culture obtained ≥ 72 h after birth and/or admission in combination with clinical signs and symptoms of infection per discretion of the attending physician. NBSI cases were further categorized into primary bloodstream infection (PBSI), CLABSI, or secondary bloodstream infection (SBSI), depending on the site of acquisition. CLABSI was defined according to the Dutch neonatal CLABSI Surveillance Criteria, with a central-line being at risk for infection if in place for > 48 h, until the day of removal or the following day (Supplementary information 1) [8]. Identification of CLABSI occurred via a (semi-)automatic surveillance system operating both retrospectively and in real-time, combining clinical data with the date of admission, blood culture results, CRP levels, and presence of central-lines. Both PBSI and SBSI were defined according to the CDC/NHSN Surveillance Definitions for Specific Types of Infection [9].

Neonates with positive blood cultures owing to common commensals as referenced in the NHSN Master Organisms list [9], including but not limited to coagulase-negative staphylococci (CoNS), were included as bacteremic cases in the presence of CRP ≥ 10 mg/L within the first 36 h after start of infection or two separate cultures. Multiple positive blood cultures from a single neonate yielding the same species with identical antibiotic susceptibility patterns were attributed to a single episode of infection if the time between positive cultures was < 7 days. We defined a new bacteremic case as such after isolation of a new organism in combination with the initiation of a new course of appropriate antibiotic treatment.

### Statistical analysis

Data are reported as mean and standard deviation (SD), median and interquartile range (IQR), or absolute number and percentages, where appropriate. Categorical data were analyzed using the chi-square test of independence or Fisher’s exact test and continuous data using the one-way analysis of variance test or Kruskal–Wallis test. Annual NBSIs were calculated as the number of infection episodes per 1000 patient-days (incidence density) and per 100 infants (cumulative incidence rate). CLABSI rates were calculated per 1000 central-line days.

To evaluate the presence of special-cause variation in incidence of NBSI and CLABSI, two Shewhart *U* charts depicting half-yearly NBSI (per 1000 patient-days) and CLABSI rates (per 1000 central-line days) were created. To quantify antibiotic use, annual rates of total days of treatment (DOT) and total length of treatment (LOT), both normalized per 1000 patient-days, were calculated. DOT represents an aggregate sum of days of exposure to each separate antibiotic agent (i.e., 2 different antibiotics administered for 2 days amount to 4 DOTs), while LOT refers to the number of days of exposure to at least one dose of any antibiotic. For Poisson-distributed count and rate data, two models, respectively with and without year added as a factor, were compared using analysis of variance. The likelihood-ratio test was used to assess if year significantly improved model fit.

Although CoNS typically account for as much as 60% of reported neonatal bloodstream infections, whether their isolation from a single-blood culture reflects true bacteremia or contamination remains a subject of debate [2, 4, 10, 11, 12]. To account for a potential over-representation of CoNS-related CLABSI, we separately estimated the annual burden of CLABSI caused by non-CoNS micro-organisms.

A multi-state Markov model was created to model transition probabilities of the following five states of NBSI progression: admission without a central-line, infected with NBSI, healthy with a central-line, death, and discharged without infection. The model was used to assess (i) transition probabilities of each defined state, and (ii) probability of acquiring an infection given presence of a central-line while admitted without having an infection on days 0, 3, 7, and 10 of admission. Data were censored at discharge. A *p*-value < 0.05 was considered statistically significant. All statistical analyses were performed using R (version 4.0.3) [13].

## Results

Between January 1st, 2012 and December 31st, 2020, 1586 very-preterm neonates were admitted to our NICU, of which 39 were excluded as a result of gestational age below 24 weeks (*n* = 1), admission for (pre)terminal care (*n* = 3), and admission duration below 24 h (*n* = 35). Demographic characteristics of the 1547 included neonates are shown in Table 1. Median gestational age was 29 weeks (IQR 27–30 weeks) and median birth weight was 1237 g (IQR 955–1516 g). Extremely-low-birth-weight infants (< 1000 g) comprised nearly one third (28.3%) of the study population. Forty-eight neonates (3.1%) were born with a major congenital anomaly whose proportion decreased over time (6.8% in 2014 to 2.3% in 2020, *p* = 0.03, data not shown). Sixty-six (4.3%) infants died during hospitalization.

**Table 1:** Neonatal baseline characteristics

Characteristic	*N* = 1,547
Sex	
Male	829 (53.6%)
Female	718 (46.4%)
Birth modus	
Vaginal	745 (48.2%)
Caesarian section	802 (51.8%)
Multiple gestation	610 (39.4%)
Gestational age (weeks)	29 [27,30]
24^0/7^–25^6/7^	140 (9.0%)
26^0/7^–27^6/7^	258 (16.7%)
28^0/7^–29^6/7^	468 (30.3%)
30^0/7^–31^6/7^	681 (44%)
Birth weight (grams)	1,237 [955, 1,516]
< 750	150 (9.7%)
750–1000	287 (18.6%)
1001–1500	699 (45.2%)
> 1500	411 (26.6%)
Apgar score at 5 min^a^	8 [7, 9]
Major congenital anomaly	48 (3.1%)
Invasive mechanical ventilation	613 (39.6%)
Duration invasive mechanical ventilation (days)	5 [2, 10]
Total ventilation days	4,895
Length of hospital stay per neonate (days)	13 [6, 29]
Total patient-days	32,055
In-hospital mortality	66 (4.3%)

Results are presented as *N*, n (%) or median [IQR]. ^a^Calculated over 1536 neonates (11 missing variables)

### Nosocomial bloodstream infection outcomes

**Fig. 1:**
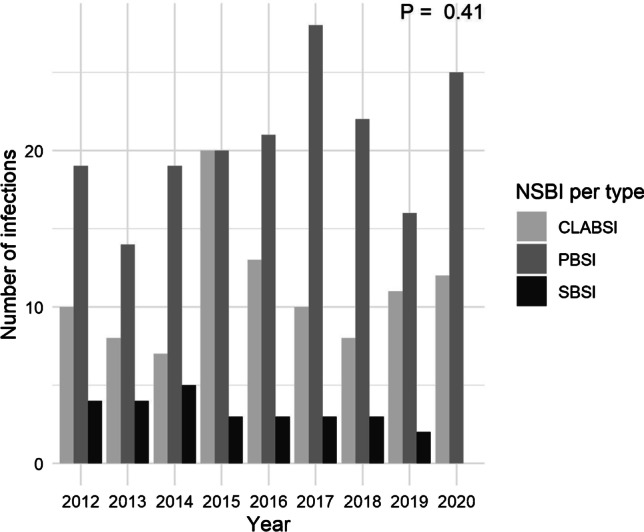
Annual number of nosocomial bloodstream infection episodes per type

Throughout the study period, a total of 292 neonates acquired 310 episodes of NBSI, 184 (59.4%) of which were classified as primary bacteremia, 99 (31.9%) as CLABSI, and 27 (8.7%) as SBSI seeded from another primary infection site (Supplementary information 2 and Fig. 1). Common primary foci for SBSIs were pneumonia, urosepsis, osteomyelitis, and necrotizing enterocolitis. Annual NBSI incidence rates varied from 8.15 to 11.5 per 1000 patient-days, with no significant differences between years (*p* = 0.75). Likewise, no differences were found between annual cumulative incidence rates of NBSI, varying between 14.2 and 27.2 per 100 infants (*p* = 0.16).

**Fig. 2:**
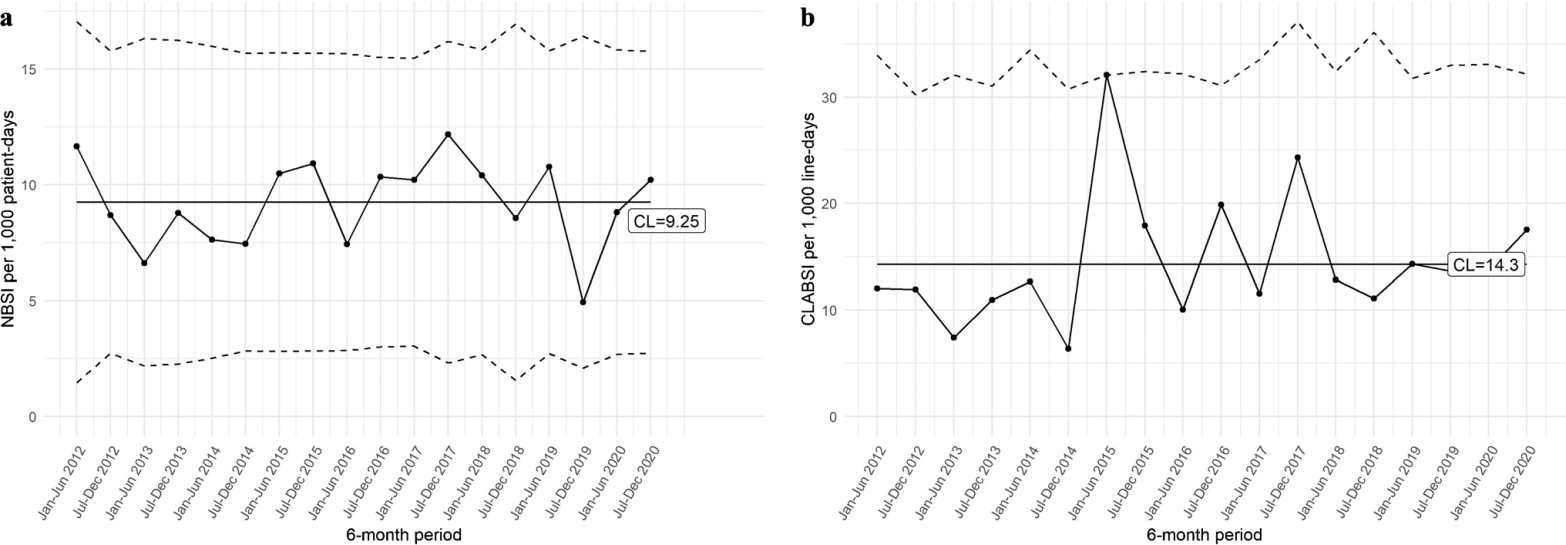
**a** and **b** *U* charts for overall central-line associated and nosocomial bloodstream infection rates per 6 months. Center line represents the central-limit (CL). Upper and lower lines represent the upper- and lower-control limits, respectively (UCL, LCL). LCL in Fig. 2b includes zero and is therefore not depicted

In order to directly monitor observed proportions and potential significant changes therein over time, *U* chart analysis depicting half-yearly NBSI rates was conducted (Fig. 2A). Over the 9-year study period, the mean incidence rate was 9.25 per 1000 patient-days. No deviations from the expected NBSI rate distribution were observed, indicating a relatively stable infection occurrence and no special cause variation.

### Central-line characteristics and CLABSI outcomes

**Fig. 3:**
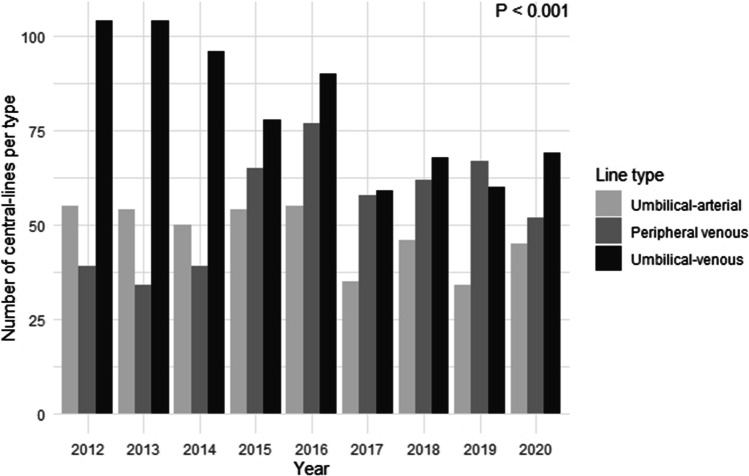
Annual number of central-lines per line type

A total of 1649 central-lines covering 7239 line-days were placed in 891 neonates (demographics shown in Table 2). The number of central-lines placed decreased significantly over time, with a relative shift towards more peripheral venous lines (Fig. 3, *p* < 0.001). Similarly, a decrease over time was found in the total number of neonates exposed to a central-line (69% in 2012 to 51% in 2020, *p* < 0.001), with a relative increase and decrease among 24^0/7^–25^6/7^ and 30^0/7^–31^6/7^ gestational age groups, respectively (Supplementary information 3). In contrast, increases were found in the median number of line-days per neonate (from 6.2 [IQR 3.8,8.4] to 7.2 [IQR 5.7,10.3], *p* = 0.002) and median age at central-line insertion (0.75 [IQR 0.47,1.04] to 0.93 [IQR 0.67,1.45], *p* < 0.001) and removal (7 [IQR 5,10] to 9 [IQR 7,12], *p* < 0.001). Central-line dwell-time was longest among the youngest infants (24^0/7^–25^6/7^ weeks’ gestation) (Supplementary information 3). Ninety-seven neonates (11%) developed at least one CLABSI, with the majority of infectious episodes occurring in infants born < 30 week’s gestation (Supplementary information 3). The mean 9-year CLABSI incidence was 14.3 per 1000 central-line days, with no significant difference between annual rates. *U* chart analysis revealed a statistically significant rise in CLABSI to 25.3 per 1000 central-line days in 2015 with subsequent spontaneous normalization, indicating the presence of special cause variation (Fig. 2B).

**Table 2:** Demographics neonates with a central-line

Year	2012	2013	2014	2015	2016	2017	2018	2019	2020	*P* value^a^
Number of neonates with a central-line	119 (69%)	120 (66%)	110 (62%)	96 (65%)	116 (62%)	75 (44%)	81 (53%)	86 (47%)	88 (51%)	** < 0.001**
Age at central-line insertion	0.75 [0.47, 1.04]	0.74 [0.53, 0.96]	0.87 [0.63, 1.04]	0.75 [0.58, 0.92]	0.90 [0.59, 1.35]	0.97 [0.68, 1.37]	0.88 [0.60, 1.09]	0.92 [0.57, 1.55]	0.93 [0.67, 1.45]	** < 0.001**
Age at central-line removal	7 [5, 10]	8 [6, 10]	8 [6, 10]	8 [6, 12]	9 [6, 12]	10 [7, 12]	9 [6, 12]	10 [7, 13]	9 [7, 12]	** < 0.001**
Line-days per neonate	6.2 [3.8, 8.4]	6.6 [4.5, 8.8]	6.8 [4.5, 8.9]	7.2 [5.0, 10.1]	6.6 [4.5, 9.2]	6.8 [4.9, 9.8]	7.4 [4.9, 9.4]	7.8 [5.8, 11.0]	7.2 [5.7, 10.3]	**0.002**
Total line-days	862	835	793	792	852	594	660	787	764	–
Neonates with CLABSI	10 (8.4%)	8 (6.7%)	7 (6.4%)	19 (20%)	13 (11%)	10 (13%)	8 (9.9%)	10 (12%)	12 (14%)	0.07
CLABSI incidence per 1,000 line-days	11.6	9.58	8.83	25.3	15.3	16.8	12.1	13.9	15.7	0.27^b^

Results are presented as *N*, n (%), median [IQR] or *N* normalized per 1000 line-days. Bold emphasis corresponds to the *p*-values which are statistically significant (i.e. *p* < 0.05)

*CLABSI*, central-line associated bloodstream infection

^a^*P* values corresponds to the chi-square test of independence and Kruskal–Wallis test, as appropriate

^b^*P* values correspond to ANOVA test of the increase in model fit by adding year to Poisson regression

### Microbial etiology of NBSI

Table 3 details the microbiological spectrum of all NBSI subtypes. Coagulase-negative staphylococci were the most frequently isolated pathogens, accounting for 66% of NBSI episodes. Among the gram-positive cocci group, methicillin sensitive *Staphylococcus aureus* was the second most common microorganism, causing 12.6% of NBSI episodes. Gram-negative species were isolated in 11% of episodes, with *Escherichia coli* dominating the SBSI group (*n* = 5, 19%).

**Table 3:** Pathogen distribution for NBSI

	CLABSI, *N* = 99	PBSI, *N* = 184	SBSI, *N* = 27
Gram-positive, *n* (%)	**89 (90%)**	**160 (87%)**	**16 (59%)**
* CoNS*	75 (75%)	125 (68%)	4 (15%)
* Staphylococcus aureus* (MSSA)	8 (7.9%)	22 (12%)	9 (33%)
* Streptococcus* spp^a^	0	2 (1.1%)	0
* Enterococcus* spp^b^	6 (5.9%)	7 (3.8%)	3 (11.1%)
* Streptococcus agalactiae*	0	4 (2.2%)	0
Gram-negative, *n* (%)	**9 (9%)**	**16 (8.7%)**	**9 (33%)**
* E. coli*	5 (5.0%)	6 (3.3%)	5 (19%)
* Enterobacter* spp	1 (1.0%)	5 (2.7%)	2 (7.4%)
* Klebsiella* spp	1 (1.0%)	2 (1.1%)	0
* Pseudomonas aerginosa*	1 (1.0%)	1 (0.5%)	1 (3.7%)
* Morganella morganii*	0	1 (0.5%)	0
* Serratia marcescens*	0	0	1 (3.7%)
* Citrobacter* spp non koseri	0	1 (0.5%)	0
* Acinetobacter* spp	1 (1.0%)	0	0
Fungi, *n* (%)	**1 (1.0%)**	**8 (4.3%)**	**2 (7.4%)**
* Candida* spp	1 (1.0%)	4 (1.6%)	1 (3.4%)
Polymicrobial, *n* (%)	0	4 (2.2%)	1 (3.7%)

Results are presented as *N* or *n* (%). Bold emphasis corresponds to the totals of the pathogen categories

*CoNS*, coagulase-negative staphylococci; *MSSA*, methicillin sensitive *Staphylococcus aureus*

^a^Encompass *Streptococcus bovis* and *Streptococcus viridans* (alpha hemolytic streptococci)

^b^Encompass *Enterococcus faecalis* and *Enterococcus faecium*

### Subgroup analysis for non-CoNS CLABSI

Supplementary information 4 shows the annual CLABSI incidence for non-CoNS related pathogens only. Twenty-four (25%) CLABSI episodes were attributable to non-CoNS related microorganisms. When CLABSI episodes caused by CoNS were assumed to represent contamination instead of true bacteremic cases, a 75% decrease (from 14.3 to 3.34 per 1000 central-line days) was seen in the risk of CLABSI. No difference was likewise found in the annual rates of non-CoNS CLABSI.

### Antibiotic consumption

**Fig. 4:**
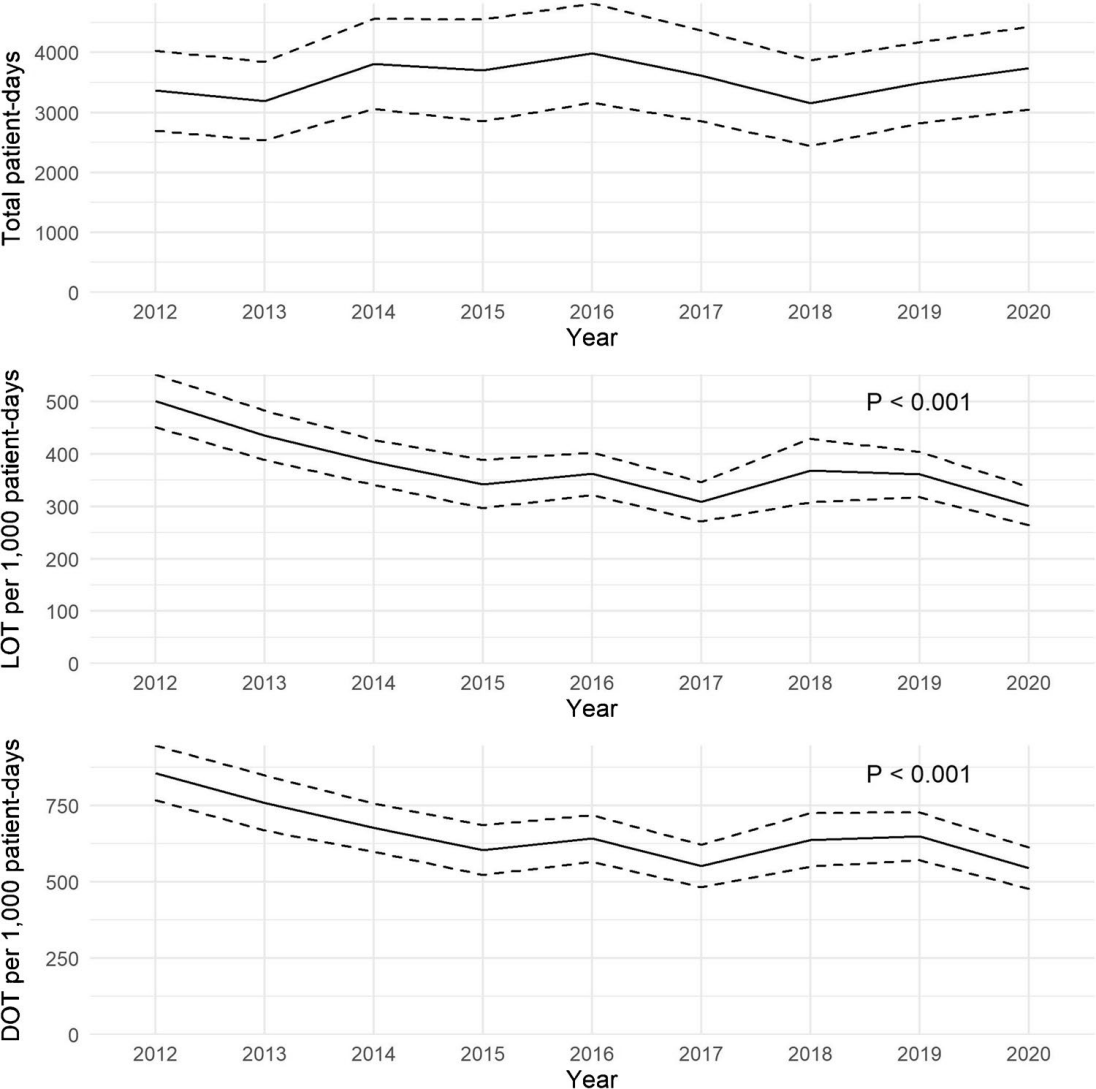
Annual number of patient-days and antibiotic consumption rates. LOT, length of therapy; DOT, days of therapy. Plots display annual mean patient-days, LOT, and DOT rates with 95% confidence intervals*. P* values correspond to ANOVA test of the increase in model fit by adding year to Poisson regression

A significant difference over time was found in the annual number of neonates treated with antibiotics ≥ 72 h after admission, with currently nearly 66% of infants receiving antibiotic therapy (Table 4). A total LOT and DOT of 11,897 and 20,953 days, respectively, were registered over the entire 9-year surveillance period. Significant differences were found in both the annual median LOT (from 5 [IQR 4, 14] to 4 [IQR 3, 8], *p* < 0.001) and DOT per neonate (9 [IQR 6, 22] to 7 [IQR 5,15], *p* < 0.001), as well as overall antibiotic exposure (LOT and DOT rates per 1000 patient-days) (Fig. 4, both *p* < 0.001).

**Table 4:** Antibiotic treatment outcomes

	2012, *N* = 173	2013, *N* = 183	2014, *N* = 177	2015, *N* = 147	2016, *N* = 186	2017, *N* = 172	2018, *N* = 153	2019, *N* = 182	2020, *N* = 173	*P* value^a^
Neonates treated with antibiotics										
< 24 h postpartum	105 (61%)	106 (58%)	106 (60%)	85 (58%)	125 (67%)	104 (60%)	85 (56%)	108 (59%)	100 (58%)	0.60
> 72 h postpartum	138 (80%)	142 (78%)	138 (78%)	118 (80%)	140 (75%)	102 (59%)	106 (69%)	126 (69%)	109 (63%)	** < 0.001**
LOT per infant	5 [4, 14]	5 [3, 9]	6 [3, 11]	6 [3, 12]	6 [3, 11]	4 [3, 8]	5 [3, 8]	5 [3, 9]	4 [3, 8]	** < 0.001**
DOT per infant	9 [6, 15]	9 [6, 16]	9 [5, 17]	11 [5, 18]	11 [5, 19]	6 [4, 13]	9 [5, 20]	9 [5, 14]	7 [5, 21]	** < 0.001**

Results are presented as *n* (%) or median [IQR] and are calculated over the total annual number of admitted neonates. Bold emphasis corresponds to the *p*-values which are statistically significant (i.e. *p* < 0.05)

*hrs*, hours; *LOT*, length of therapy; *DOT*, days of therapy

^a^*P* value corresponds to the chi-square test of independence and Kruskal–Wallis test, as appropriate

### Multi-state model

Both insertion and maintenance practices are essential in the prevention of CLABSI, particularly for central-lines that remain in place for prolonged periods of time [14]. Although consensus regarding optimal central-line duration remains absent, there is concern that increasing dwell-time increases the risk of CLABSI. Therefore, to determine the risk of infection over time in patients with and without a central-line, a multi-state model was created.

Probabilities over time of being in a particular state conditional on the initial state are illustrated in Supplementary information 5. On day 7 of admission, the probability of acquiring an infection is approximately 7.5%, with a peak of 8% on day 11. A steady increase in central-line placement is observed from day 0 of admission onwards, with the highest probability of central-line use on day 5 (24%), stabilizing at 1–2% by day 14. Total predicted in-hospital mortality throughout admission is approximately 3%.

Multistate modeling was likewise used to dynamically predict the association between admission duration, given a central-line, and risk of NBSI (Supplementary information 6). The probability of acquiring an NBSI, given presence of a central-line and no (previous) infection, decreases from 16% on day 3 to approximately 6% on day 10. This decrease in infection risk is accompanied by a higher probability of discharge over time, as corroborated in Supplementary information 5 by the steady transition from “healthy with a central-line” to “healthy and discharged.”

## Discussion

This study was designed to evaluate changes in incidence of NBSI, causative pathogens, and antibiotic exposure in preterm infants over a 9-year surveillance period to better inform ways to achieve reductions in infection rates. We found that 19% of preterm neonates acquired one or more episodes of NBSI and no significant changes in annual infection rates over time. Despite the reduction in number of neonates with a central-line, duration of central-line use increased significantly. The burden of CLABSI was considerable, with an incidence rate of 14.3 per 1000 central-line days including a notable yet transient increase in 2015. Under the assumption that non-CoNS CLABSI are true bacteremic cases, a 75% decrease in the risk of CLABSI was observed. Moreover, the probability of acquiring an infection is the highest in the first 1–2 weeks of life. CoNS was the most frequent cause of NBSI. Finally, a significant decrease over time was observed in overall antibiotic consumption.

Comparison with previous surveillance studies, reporting infection rates between 0.68 and 18.1 per 1000 central-line days [16, 19, 20, 21, 22], shows our CLABSI incidence to be at the higher end of the spectrum which may be explained by at least three factors. First, the significant variability in case definitions used by researchers to confirm the diagnosis of CLABSI not only results in remarkable heterogeneity in incidence rates but also compromises efforts towards benchmarking infection outcomes. Similarly, studies use various definitions of CLABSI consisting of full or partial adaptations of well-known definitions established by large surveillance networks [9, 17], without further specification for the neonatal population. By diagnosing CLABSI events using the Dutch neonatal surveillance consensus criteria via a highly-sensitive semi-automated system, we were able to circumvent issues related to the validity and sustainability of infection reporting. Second, difficulties inherent to distinguishing true bacteremia from culture contaminants may have resulted in an overestimation of our infection rates, especially considering the employment of a single-blood culture policy in the Netherlands for confirmation of CoNS CLABSI as opposed to the multiple-blood specimen policy applied in other countries. Our subgroup analysis showed that the burden of CLABSI in our NICU is predominated by CoNS, with a substantial decrease in the risk of CLABSI to 3.43 per 1000 central-line days after the exclusion of CoNS microorganisms. Furthermore, positive cultures sampled from central-lines may be caused by catheter colonization, leading to false infection source adjudication. Third, differences in reported incidences may be partially attributed to the varying composition of central-line insertion and maintenance bundles and their inconsistent execution, even if the optimal composition of bundle elements remains unknown. Even though the previously mentioned factors hinder accurate comparison of our results with those from previous studies, we can still conclude that further efforts for improvement are needed, considering CLABSIs are a preventable disease which reasonably compels (near) complete elimination.

Despite the overall reduction in central-line use, we observed no concomitant decrease in CLABSI rates, which is suggestive of a relative increase in CLABSI episodes. Although no clear cause for the observed increase in 2015 could be identified, a potential influencing factor is the slight increase in number of extremely premature (24–25 weeks GA) infants, whom typically necessitate central-lines for a longer period of time and thereby carry the highest infection risk. Over time, central-line use increased by a median of 1 day, which may be the result of relative conservative feeding-advancement practices. In addition, infection risk was the highest during the first 6 days after central-line placement with a relative stabilization thereafter, suggesting that insertion rather than maintenance practices may be more critical in preventing CLABSI. Establishing consistent surveillance practices through standardized interinstitutional collaboration, and further limiting central-line use and dwell-time, perhaps through the adoption of more aggressive feeding protocols, and with special attention towards the most vulnerable infants, are essential next steps towards achieving and maintaining low CLABSI rates.

In our study, PBSI was the most common NBSI subtype, accounting for approximately 60% of all episodes. Although the exact source of these bacteremias is unknown, a plausible cause may be peripheral intravenous catheters (PIVs) which are commonly used in the NICU despite the high incidence (up to 80%) of complications such as obstruction, extravasation, and phlebitis [23]. Their relatively short-term patency (15–54 h) often results in a high rate of new insertion attempts, increasing the risk for CoNS-related bacteremia [15]. This is corroborated by our finding that 68% of PBSI episodes were CoNS-related. This bacteremia type should therefore be considered device-associated and targeted by prevention efforts. Unfortunately, it remains difficult to decipher what the true source of infection is in infants with both a central-line and PIV, indicating that more evidence is needed to assess the degree to which PIVs contribute to NBSI.

Although no significant decrease was observed in the number of NBSI over time, our findings did reveal a significant decrease in antibiotic use, perhaps as result of improved hospital-wide antibiotic stewardship efforts, including a reduction in antibiotic treatment duration from 7 to 3 days for an uncomplicated CoNS infection in 2015, as well as weekly meetings with infectious disease experts and discontinuation of antibiotic therapy after 48 h for culture-negative sepsis in combination with the absence of clear signs of infection in 2017. Data from other studies explicitly quantifying antibiotic consumption in neonates remains incomplete, yet our results complement those from a previous study which found a median DOT of 8 days for laboratory-confirmed BSI among very-low-birth-weight infants admitted to the NICU [18]. Given the incessant uncertainty surrounding antibiotic discontinuation as a result of diagnostic challenges within this population, increased efforts targeting specific prescribing behaviors may be useful for attaining further reductions in antibiotic exposure.

### Strengths and limitations

A major strength of our study is that it confirmed each infection case according to strict and in part standardized definitions. CLABSI cases were identified via a (semi)automated surveillance method using electronic patient, microbiology, and laboratory data, thereby providing a solid data base for research that allows the generation of high-quality results while helping to improve the capacity for continued monitoring. Furthermore, these results are presented as a longitudinal, evidence-based benchmark from which variations in practice and areas for improvement can be conveniently deduced. Apart from its retrospective nature, a limitation of this study is that we were unable to reliably attribute bacteremic cases to PIVs due to lack of available data. Moreover, although information regarding maternal GBS status, intrapartum antibiotic exposure, and duration of ruptured membranes may be associated with a higher risk of (late-onset) infection, these data were unavailable at the time of analysis. Future planned work will aim to appropriately distinguish central-line from PIV-attributed infections as well as evaluate the role of additional (prenatal) factors in the risk of HAI. Lastly, although being a competing risk for central-line dwell-time, we were unable to adjust for additional covariates such as gestational age in our multistate model due to the large number of transition states and limited number of included patients.

## Conclusion

In this study, we provide a detailed epidemiological overview of various neonatal infection entities among preterm infants whose high rates underscore the importance of this serious complication. While rates of NBSI, especially those associated with a central-line, were significantly higher than those reported in other studies, our results provide up-to-date information on the burden of HAI which can be used for (re)evaluation of existing local practices, inter-hospital rate comparison, and implementation of quality improvement initiatives. Effective measures to reduce NBSI, including limiting the use of invasive devices, rigorous bundle adherence, staff education, and ongoing identification of site-specific factors that drive sustained success for improvement, are urgently needed.

## Supplementary Information

Below is the link to the electronic supplementary material.

Supplementary file1 (DOCX 1676 KB)

## Data Availability

The datasets generated during and/or analyzed during the current study are not publicly available due to privacy and ethical concerns, but are available from the corresponding author upon reasonable request.
