# Supplementary material for: A longitudinal analysis of nosocomial bloodstream infections among preterm neonates
Source: Eur J Clin Microbiol Infect Dis. 2022 Sep 30;41(11):1327–36. doi: 10.1007/s10096-022-04502-8 (PMC9556429; doi:10.1007/s10096-022-04502-8)

**Supplementary Information 1.** Dutch Neonatal CLABSI Surveillance Criteria (8)


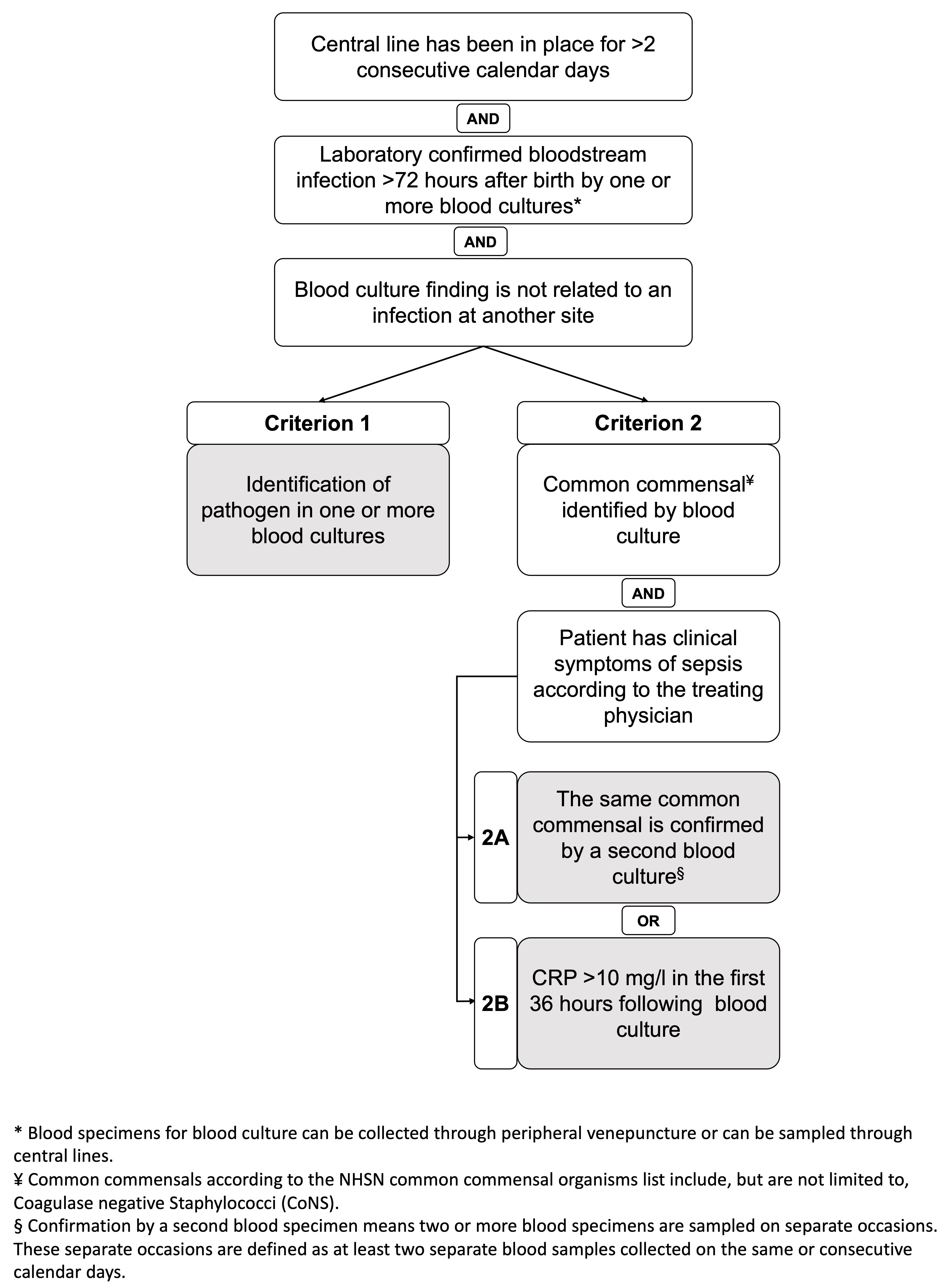


**Supplementary Information 2.** Annual Number of Neonates with NBSI

**
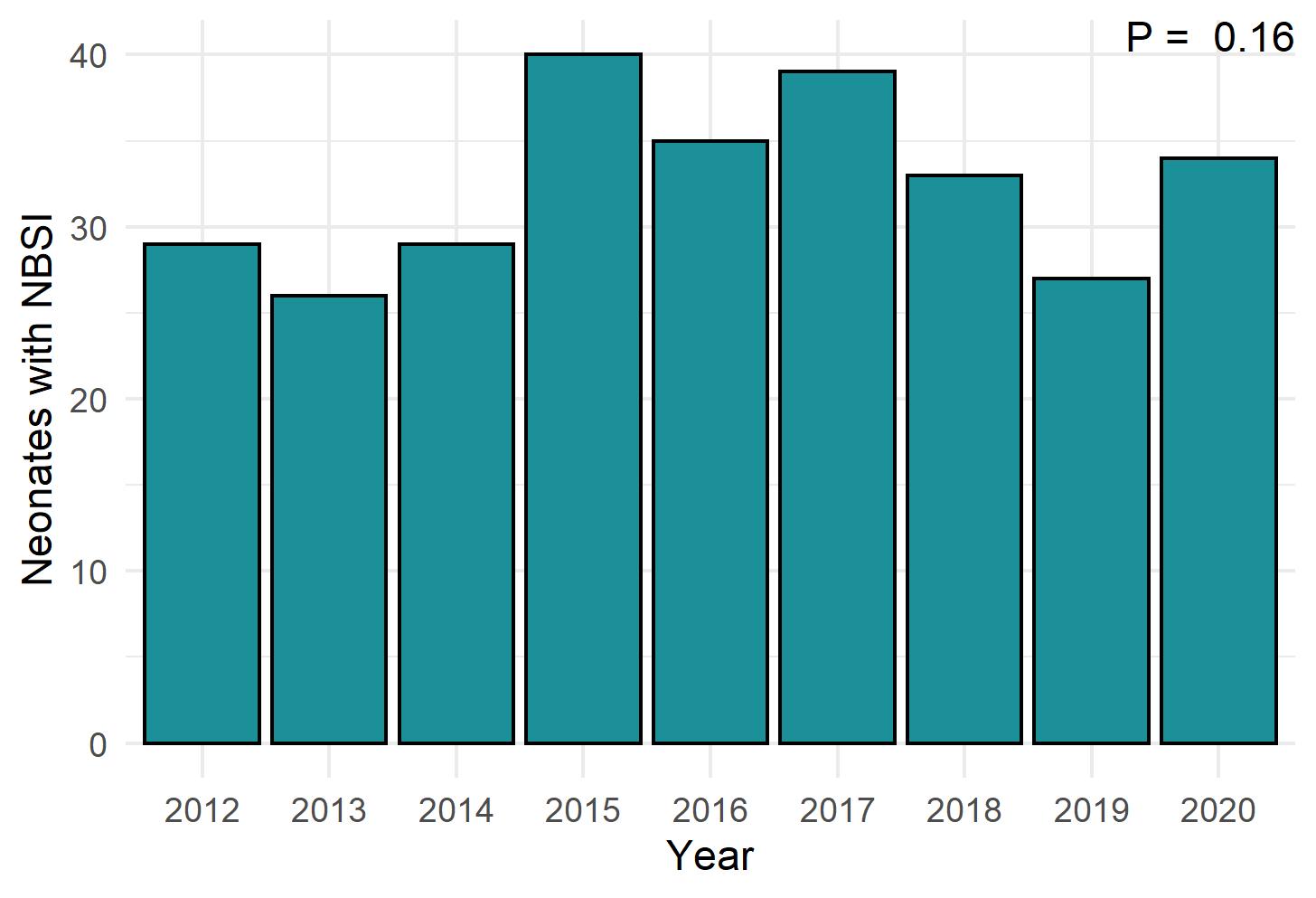
**

| **Supplementary Information 3.** Demographics Neonates with a Central-line Stratified According to Gestational Age | | | | | | | | | |
| --- | --- | --- | --- | --- | --- | --- | --- | --- | --- |
| **Year** | **2012** | **2013** | **2014** | **2015** | **2016** | **2017** | **2018** | **2019** | **2020** |
| Number of neonates with a central-line | 119 (69%) | 120 (66%) | 110 (62%) | 96 (65%) | 116 (62%) | 75 (44%) | 81 (53%) | 86 (47%) | 88 (51%) |
| 24^0/7^- 25^6/7^ wks | 15 (11%) | 9 (6.8%) | 12 (9.0%) | 10 (7.5%) | 16 (12%) | 14 (11%) | 22 (17%) | 20 (15%) | 15 (11%) |
| 26^0/7^- 27^6/7^ wks | 28 (12%) | 23 (10%) | 25 (11%) | 30 (13%) | 26 (11%) | 27 (12%) | 24 (10%) | 18 (7.8%) | 29 (13%) |
| 28^0/7^- 29^6/7^ wks | 42 (12%) | 56 (17%) | 51 (15%) | 34 (10%) | 44 (13%) | 24 (7.1%) | 23 (6.8%) | 32 (9.5%) | 31 (9.2%) |
| 30^0/7^- 31^6/7^ wks | 34 (18%) | 32 (17%) | 22 (12%) | 22 (12%) | 30 (16%) | 10 (5.2%) | 12 (6.3%) | 16 (8.4%) | 13 (6.8%) |
| Line-days per neonate | 6.2 [3.8, 8.4] | 6.6 [4.5, 8.8] | 6.8 [4.5, 8.9] | 7.2 [5.0, 10.1] | 6.6 [4.5, 9.2] | 6.8 [4.9, 9.8] | 7.4 [4.9, 9.4] | 7.8 [5.8, 11.0] | 7.2 [5.7, 10.3] |
| 24^0/7^- 25^6/7^ wks | 7 (4,14) | 9 (6,11) | 12 (6.9) | 8 (7.16) | 9 (7,12) | 5 (3,9) | 9 (6,11) | 10 (7,16) | 10 (8,16) |
| 26^0/7^- 27^6/7^ wks | 6.6 (4.6,10.1) | 7.1 (5.1,9.9) | 7.4 (5.0,8.7) | 8.9 (6.9,11.3) | 7.0 (5.9,10.7) | 7.7 (6.4,10.8) | 7.9 (5.0.1,9) | 7.3 (5.9,8.8) | 7.1 (5.2,10.3) |
| 28^0/7^- 29^6/7^ wks | 6.7 (4.2,8.7) | 6.8 (4.9, 8.3) | 7.1 (5.0,9.0) | 6.3 (4.6,9.6) | 6.1 (4.0, 7.5) | 6.3 (5.3, 8.9) | 6.9 (4.0, 8.8) | 6.9 (5.5, 10.8) | 6.9 (5.3,10.2) |
| 30^0/7^- 31^6/7^ wks | 4.7 (2.4,6.4) | 5.3 (3.5,8.4) | 4.1 (2.1,6.8) | 4.9 (3.4,7.8) | 5.8 (3.9,7.6) | 5.0 (3.3,7.3) | 5.1 (4.8,7.6) | 7.6 (4.9,8.8) | 6.3 (5.1,7.4) |
| Total line-days | 862 | 835 | 793 | 792 | 852 | 594 | 660 | 787 | 764 |
| 24^0/7^- 25^6/7^ wks | 156 | 80 | 158 | 110 | 152 | 95 | 229 | 244 | 189 |
| 26^0/7^- 27^6/7^ wks | 214 | 185 | 163 | 291 | 240 | 236 | 186 | 142 | 239 |
| 28^0/7^- 29^6/7^ wks | 280 | 386 | 370 | 266 | 275 | 205 | 174 | 262 | 254 |
| 30^0/7^- 31^6/7^ wks | 211 | 184 | 103 | 124 | 184 | 57 | 71 | 137 | 82 |
| Neonates with CLABSI | 10 | 8 | 7 | 19 | 13 | 10 | 8 | 10 | 12 |
| 24^0/7^- 25^6/7^ wks | 0 (0%) | 0 (0%) | 2 (29%) | 5 (26%) | 6 (46%) | 2 (20%) | 1 (12%) | 6 (60%) | 4 (33%) |
| 26^0/7^- 27^6/7^ wks | 3 (30%) | 2 (25%) | 2 (29%) | 10 (53%) | 5 (38%) | 3 (30%) | 5 (62%) | 1 (10%) | 2 (17%) |
| 28^0/7^- 29^6/7^ wks | 4 (40%) | 6 (75%) | 2 (29%) | 2 (11%) | 2 (15%) | 4 (40%) | 1 (12%) | 2 (20%) | 5 (42%) |
| 30^0/7^- 31^6/7^ wks | 3 (30%) | 0 (0%) | 1 (14%) | 2 (11%) | 0 (0%) | 1 (10%) | 1 (12%) | 1 (10%) | 1 (8.3%) |
| CLABSI incidence per 1,000 line-days | 11.6 | 9.58 | 8.83 | 25.3 | 15.3 | 16.8 | 12.1 | 13.9 | 15.7 |
| 24^0/7^- 25^6/7^ wks | 0.00 | 0.00 | 12.7 | 54.4 | 39.4 | 21.0 | 4.36 | 28.6 | 21.1 |
| 26^0/7^- 27^6/7^ wks | 14.0 | 10.8 | 12.3 | 34.4 | 20.8 | 12.7 | 26.9 | 7.05 | 8.38 |
| 28^0/7^- 29^6/7^ wks | 14.3 | 15.5 | 5.41 | 7.51 | 7.26 | 19.5 | 5.73 | 7.63 | 19.7 |
| 30^0/7^- 31^6/7^ wks | 14.2 | 0.00 | 9.74 | 16.1 | 0.00 | 17.4 | 14.1 | 7.38 | 12.2 |
| Results are presented as N, n (%), median [IQR] or N normalized per 1,000 line-days.  wks, weeks; CLABSI, central-line associated bloodstream infection | | | | | | | | | |

| **Supplementary Information 4.** Sensitivity Analyses for Non-CoNS-related CLABSI | | | | | | | | | | | |
| --- | --- | --- | --- | --- | --- | --- | --- | --- | --- | --- | --- |
| **Year** | **2012** | **2013** | **2014** | **2015** | **2016** | **2017** | **2018** | **2019** | | **2020** | ***P* value**^a^ |
| Non-CoNS CLABSI episodes | 3 | 0 | 2 | 2 | 5 | 2 | 3 | | 4 | 3 | – |
| Non-CoNS CLABSI incidence per 1,000 line-days | 3.42 | 0.00 | 2.46 | 2.23 | 5.51 | 3.29 | 4.49 | | 4.85 | 3.79 | 0.42 |
| Results are presented as N or N normalized per 1,000 line-days.  CoNS, coagulative negative staphylococci; CLABSI, central-line associated bloodstream infection.  ^a^ *P* value corresponds to ANOVA test of the increase in model fit by adding year to Poisson regression. | | | | | | | | | | | |

**Supplementary Information 5.** Transition Probabilities According to Different States


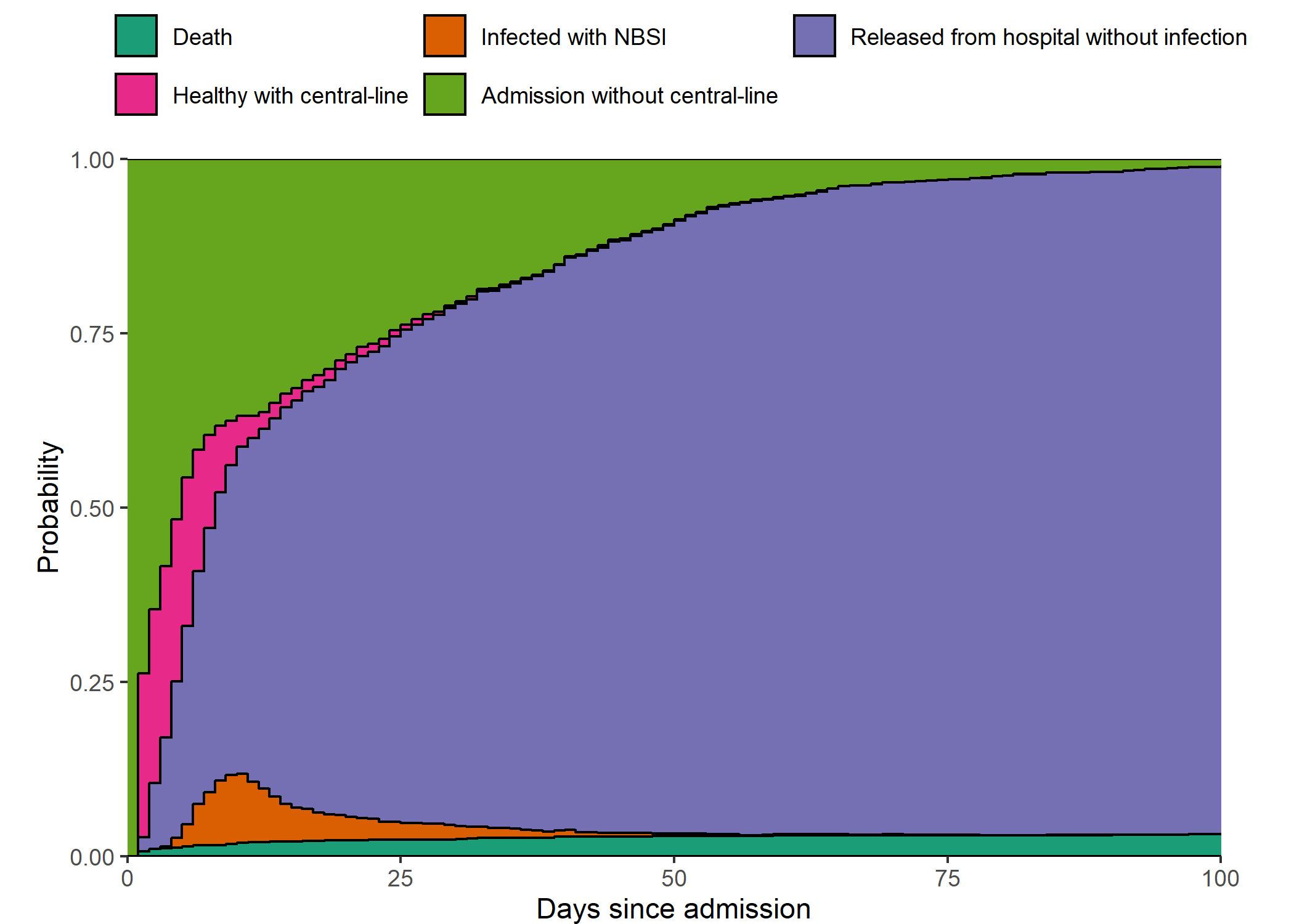


**Supplementary Information 6.** Overall NBSI Risk According to Admission Duration Given a Central-line


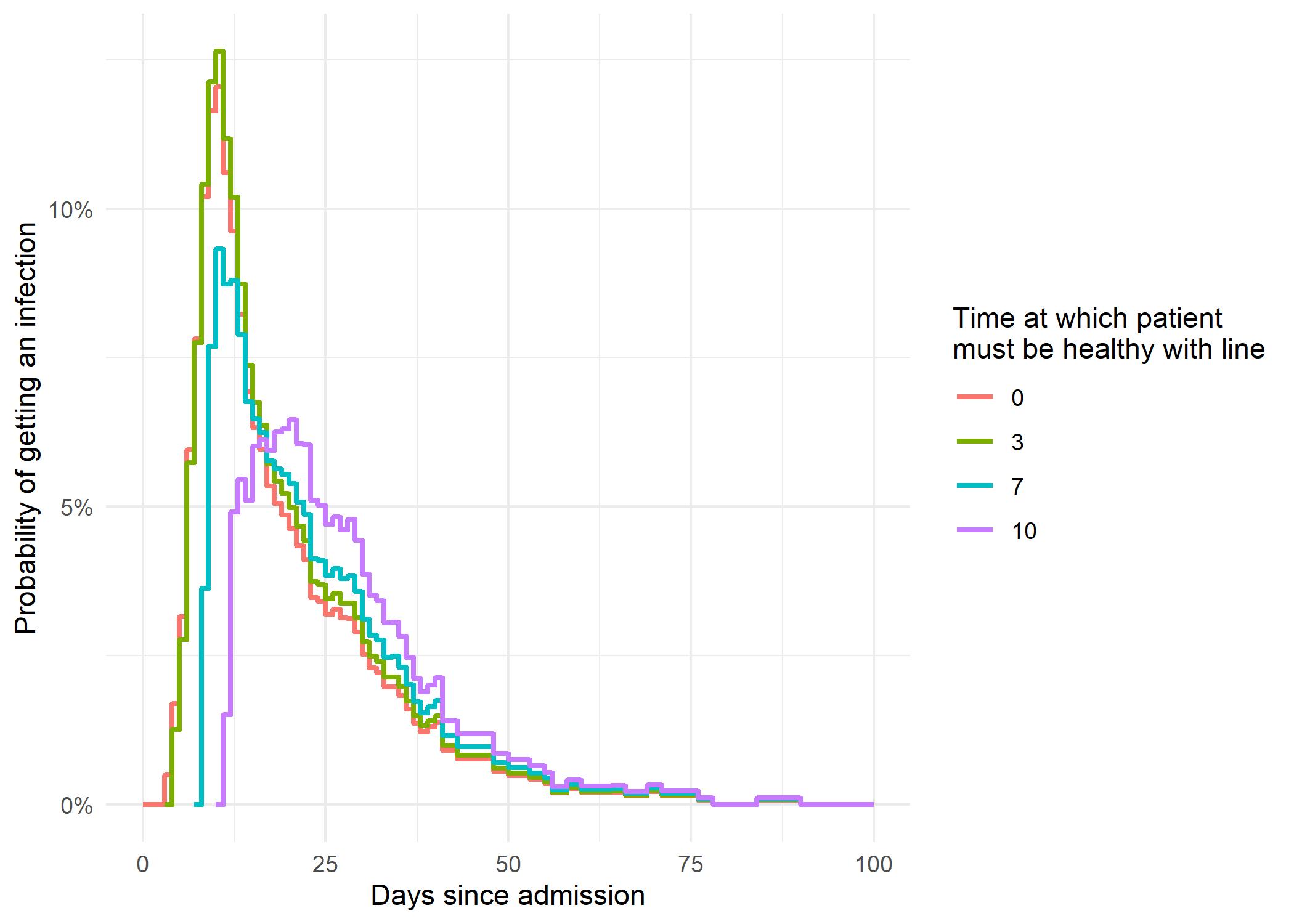

Supplement: Supplementary file 1 — Supplementary file1 (DOCX 1676 KB) [file 10096_2022_4502_MOESM1_ESM.docx]
